# *Baltorussus* Total Makeover: Rejuvenation and Sex Change in an Ancient Parasitoid Wasp Lineage

**DOI:** 10.1371/journal.pone.0098412

**Published:** 2014-06-02

**Authors:** Lars Vilhelmsen, Dominique Zimmermann

**Affiliations:** 1 Biosystematics, Natural History Museum of Denmark, Copenhagen, Denmark; 2 2nd Zoological Department, Natural History Museum Vienna, Vienna, Austria; Penn State University, United States of America

## Abstract

The Orussidae is a small and rare but phylogenetically important family of parasitoid wasps. The fossil record of the family is also very poor. *Baltorussus velteni* was described from Baltic amber from an allegedly female specimen. This and another recently discovered specimen are examined with microCT scanning and standard microscopy. We reveal that both the holotype and the new specimen are actually males. Furthermore, the results of the microCT scanning allow us to integrate the fossils in a morphological data set assembled for extant Orussidae. Phylogenetic analyses consistently retrieve *Baltorussus* as a separate basal lineage within the crown group, whereas two Cretaceous fossils are placed as stem group orussids and a Dominican amber fossil in an extant genus. Based on the positions of the fossils, we estimate that the extant Orussidae radiated in the mid-Cretaceous (approx. 100 Ma ago). This is considerably younger than a previously suggested Early Jurassic date (180 Ma ago), which was primarily based on biogeographic evidence.

## Introduction

Fossils have traditionally played a crucial role when interpreting the evolutionary history of various groups of organisms. Vertebrate fossils (e.g., the proto-bird *Archaeopteryx*) have figured prominently in phylogenetic hypotheses ever since the publication of Darwin's *The Origin of Species* promoted broader acceptance of the occurrence of evolution in organisms over deep time. In contrast, it is only comparatively recently that sufficient information has accumulated to fully realize the potential of fossils to interpret insect evolution [1]. However, with the implementation of improved methods for handling fossils in phylogenetic analyses [2], fossils are now routinely employed to calibrate age estimates of splitting events within insect phylogenies. In this paper, we demonstrate how the incorporation of a recently described fossil in the phylogeny of a family of little known parasitoid wasps, the Orussidae, leads to drastic re-evaluation of the timing of its diversification.

The Orussidae is an obscure group of parasitoid wasps that nonetheless occupy a pivotal position in the early evolutionary history of the Hymenoptera, one of the most diverse and economically important insect orders. In most recent comprehensive treatments of hymenopteran phylogeny [3], [4], the Orussidae are placed as the sister group to the Apocrita, the latter comprising the majority of the Hymenoptera including such well known groups as ants, bees and hornets as well as a multitude of parasitoid lineages. The Orussidae are parasitoids of woodboring insect larvae, typically of jewel beetles or longhorn beetles. This is a lifestyle that is probably very similar to that of the common ancestor of all carnivorous wasps, estimated to have lived 200+ Ma ago [2]. Orussidae is thus a crucial group when interpreting the transition from the herbivorous to the carnivorous lifestyle early in hymenopteran evolution [5]. Furthermore, the female orussids display remarkable adaptations to targeting host larvae living inside wood. The female apparently operates a vibrational sounding system to detect potential hosts. It generates vibrations in the wood by tapping it with the modified tips of the antennae; the vibrations are then picked up by the elongate basitarsus (the proximal part of the ‘foot’) of the fore leg and conveyed to an enlarged sensory unit, the subgenual organ, inside the swollen and subdivided tibia, where they are transduced into nerve impulses [6].

The Orussidae is a small taxon, currently with approx. 90 described extant species [7]. They occur in all major biogeographic regions, but are rarely encountered. Their fossil record is correspondingly poor, a total of four fossil taxa being unequivocally assigned to the family, all described from amber inclusions (age estimates from [1]): *Mesorussus taimyrensis* Rasnitsyn, 1977 ([8]; Siberian Taimyr amber, 95 Ma), *Minyorussus luzzii* Basibuyuk et al., 2000 ([9]; New Jersey amber, 90 Ma), *Baltorussus velteni* Schedl, 2011 ([10]; Baltic amber, 44 Ma), *Ophrynopus peritus* Engel, 2008 ([11]; Dominican amber, 17–20 Ma). Vilhelmsen [12] analyzed the two Cretaceous fossils with the extant Orussidae. The fossils were invariably placed in the stem group of the family, although the relative positions of the fossils were unresolved. In contrast, *Ophrynopus peritus* was described in an extant genus [11]; this has been corroborated by later analyses [7]. Based on the placement of the Cretaceous fossils and in particular the biogeographic patterns within the Orussidae, it was concluded that the earliest splitting events within the extant lineages of the family probably took place at least 180 Ma ago [12].

Some Upper Jurassic compression fossils from the Karatau Formation, Kazakhstan (152–158 Ma) collectively referred to as the Paroryssidae have been considered to be ‘ancestral’ to the Orussidae [13], [14]. These fossils share some wing reduction characters with extant orussids (and to some extent Apocrita), but they miss some of the characteristics of the Orussidae. Paroryssidae do not have an ocellar corona (a circle of small cuticular teeth on the top of the head) and they have a comparatively long external ovipositor whereas that of extant Orussidae is elongate but entirely concealed within the body when not in use [6]. The analyses of Ronquist et al. [2] included two paroryssid taxa, *Praeoryssus* and *Paroryssus*, as well as *Mesorussus*; these fossil taxa were retrieved as successive sister groups to *Orussus*, the only extant orussid included in the analyses.

**Figure 1:**
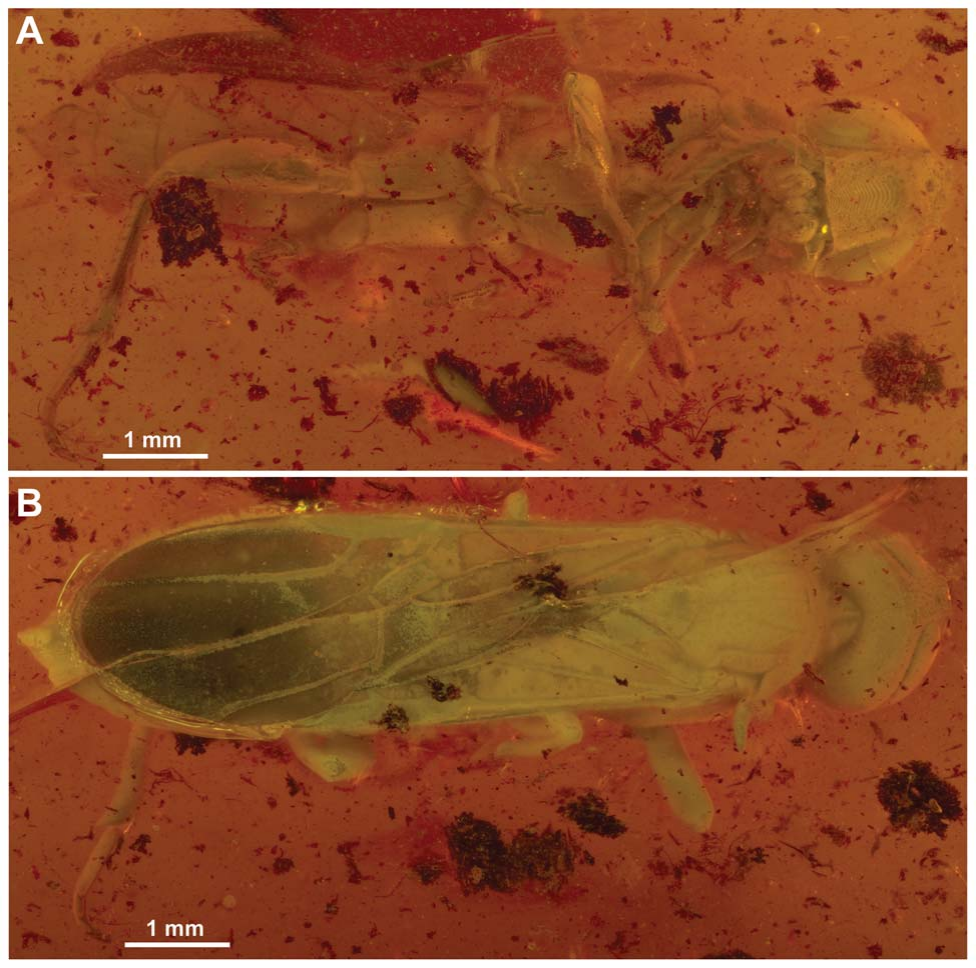
*Baltorussus velteni*, habitus holotype (NHMW 2014/0110/0001). (A) oblique ventral view; (B) dorsal view.

*Baltorussus velteni* is the first known orussid from Baltic amber. It was recently described from a single, allegedly female specimen [10]. However, the description was done without reference to recent publications on orussid systematics [12], [15], and no attempt was made to place the fossil in a phylogenetic context. Furthermore, the investigation of the fossil was carried out with only a dissection microscope. The holotype of *Baltorussus* is partly obscured by a whitish film covering most of the body and by cracks in the amber matrix surrounding the inclusion (Fig. 1).

**Figure 2:**
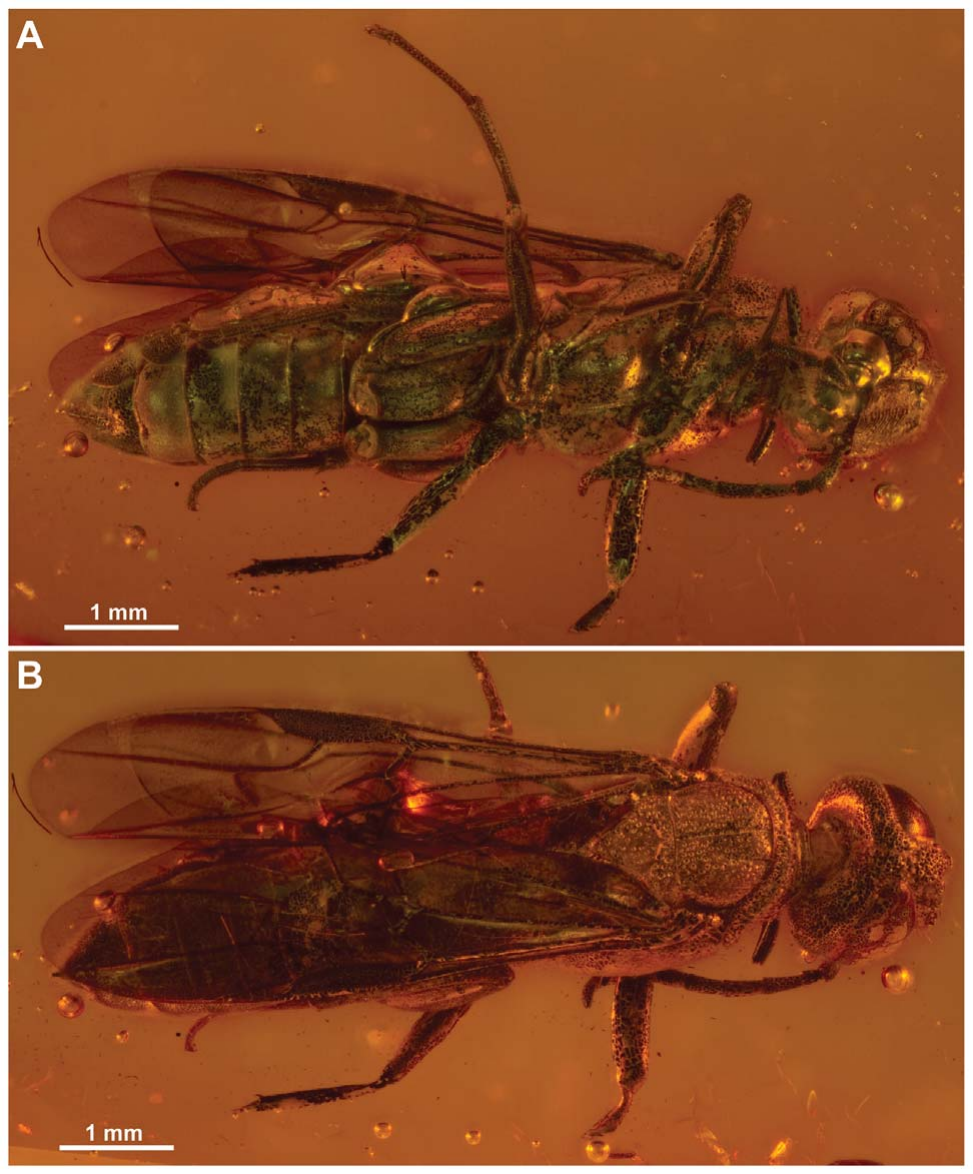
*Baltorussus velteni*, habitus non-type (NHMW 2014/0191/0001). (A) ventral view; (B) dorsal view.

Since the original description, a second specimen of Orussidae from Baltic amber has been discovered which we also identify as *Baltorussus velteni*. This specimen is better preserved (Fig. 2) and we use this and the more detailed investigation of the holotype to expand the original description and re-interpret some of the features previously reported. Furthermore, by combining observations from standard microscopy and micro-CT we are able to score enough characters for the data set initially assembled for extant Orussidae [15] to confidently place *Baltorussus* within the phylogeny of the family. Finally, we discuss the implications that the placement of *Baltorussus* has for the interpretation of the character evolution, biogeographic history and timing of the radiation of Orussidae.

## Materials and Methods

### Ethics statement

No permits were required for the described study, which complied with all relevant regulations.

### Material examined

Holotype, male; Baltic amber. Deposited in the Natural History Museum Vienna (NHMW), Dept. of Geology and Paleontology, specimen ID number: 2014/0110/0001. Original description Schedl (2011 [10]): p. 33, figs 1–2).

Additional male; Baltic amber. Deposited in the Natural History Museum Vienna (NHMW), Dept. of Geology and Paleontology, specimen ID number: 2014/0191/0001.

### Microscopy and imaging

The amber specimens were initially examined with a Leica M205C dissection microscope. They were imaged while being immersed in maple syrup. Images were captured with a Visionary Digital imaging setup with flash lighting and P-51 Camlift Driver v.2.6.1 to control the camera. Images were stored in Adobe Lightroom 2 and composite images were compiled from stacks with the software Zerene Stacker v. 1.04 by implementing the Pyramidal stacking method (PMax).

### MicroCT-scanning

**Figure 3:**
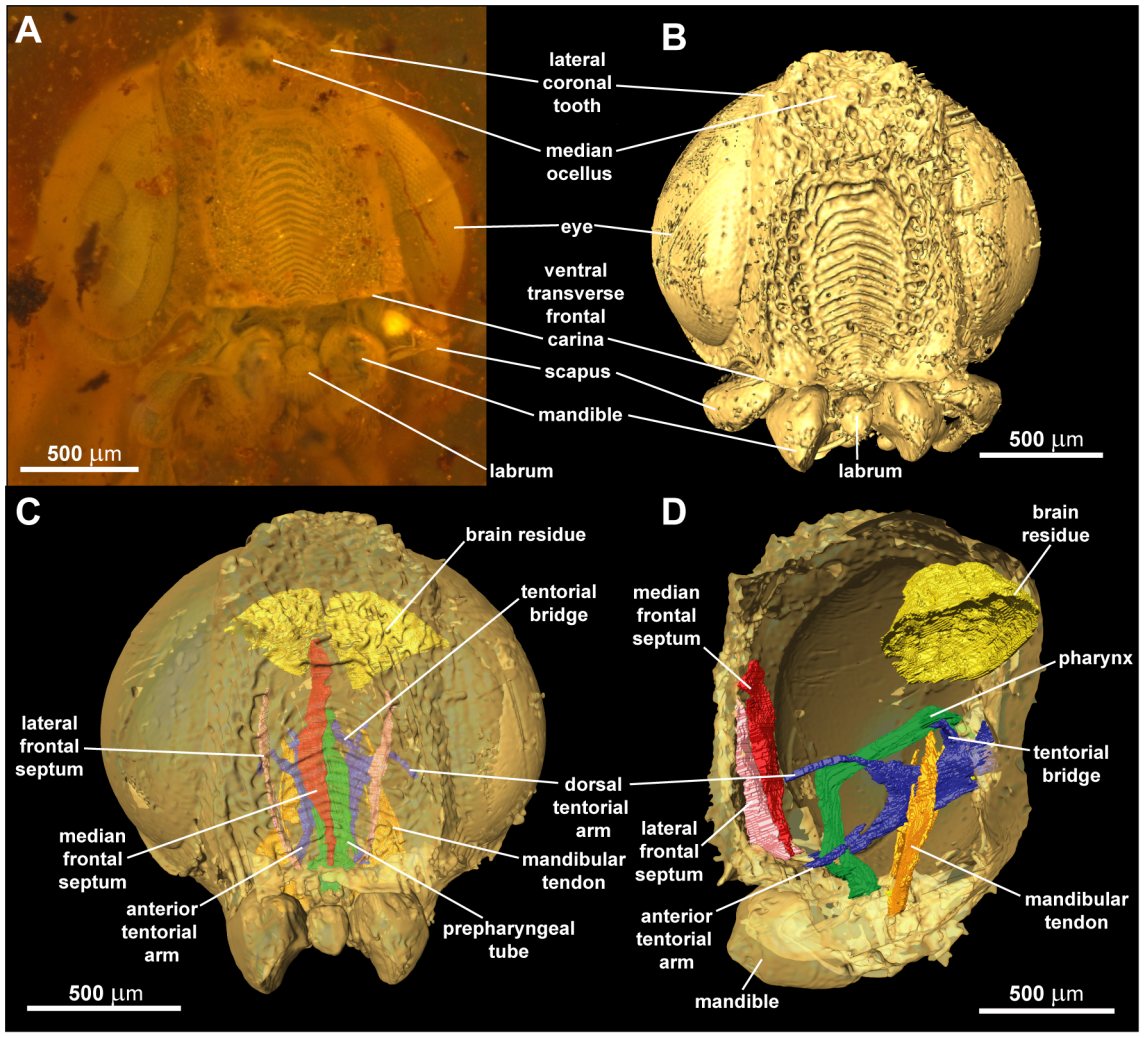
Head of *Baltorussus velteni*, holotype (NHMW 2014/0110/0001). (A) anterior view, brightfield, (B) anterior view, surface rendering, (C) anterior view, 3D reconstruction, (D) lateral view, 3D reconstruction.

The holotype of *Baltorussus velteni* was imaged with an Xradia MicroXCT x-ray microtomography system (University of Vienna, Department of Theoretical Biology). The microCT data were reconstructed with 2×2 pixel binning to reduce noise and file size, and reconstructed volume images were exported as a PNG image stack which is available for download at Morphosource (http://morphosource.org) as media file 2371. The software Amira 5.4.3 was used for 3D-visualization and analysis of the data. For the surface renderings (e.g., Fig. 3B) the image stack was cropped with the crop editor to eliminate the surface of the amber piece as far as possible. Additionally the *obliqueSlice* function was used to blank disturbing surfaces temporarily. The surface renderings were generated with the *isosurface* function. For the volume renderings (e.g., male genitalia, see below) the area of the internal genitalia was segmented. A new volume dataset was generated from the original data and the label data by using the *arithmetic* function [16]. Subsequently the *voltex* function was used to visualize the structures. For the reconstructions (e.g., Fig. 3C, D) the structures were segmented manually with the *brush* tool and visualized with the *surfaceGen* and *surfaceView* functions.

### Phylogenetic/dating analyses

The two *Baltorussus* specimens were scored for the morphological character set for Orussidae presented in [7], an update of the data matrix from Vilhelmsen [15]. The dataset was assembled in Mesquite [17]. Of the 169 characters included, *Baltorussus* could be scored for 98; eight were inapplicable and 63 had to be scored as unknown as the character in question could not be observed either because it was obscured by other body parts or because it was sex specific. In total, 58% (63% if inapplicable characters are included) of the characters could be scored for *Baltorussus*, a high percentage for a fossil hymenopteran taxon (compare with table 2 in [2]; this was a different data set which included a number of characters dealing with internal anatomy not observable in fossils). The data set is available from FigShare (http://dx.doi.org/10.6084/m9.figshare.951964). As was the case for Vilhelmsen [12], we did not include the Paroryssidae in the data set. Unlike the amber fossils included, Paroryssidae are comparatively poorly preserved compression fossils that can only be scored for very few of the characters included here, and it would be difficult to place them confidently in the phylogeny.

The data set was analyzed in TNT 1.1. [18] with the following characters treated as additive: 12, 19, 24, 31, 34, 35, 40, 46, 65, 66, 70, 75, 77, 87, 96, 103, 104, 111, 113, 114, 119, 124, 125, 126, 137, 146, 147, 149, 152, 156, 157, 159, 160, 164, and 167. Space for 1 000 000 trees was reserved in the memory. Analyses were performed under equal and implied weights. For implied weighting [19], the concavity constant K was set in turn to 3, 6, 8 and 10–20. For each weighting scheme, traditional analyses with 10 000 replications and TBR saving 100 trees per replication were conducted. All analyses were run with collapsing rule 1. The root was *Urocerus*. To calculate the Bremer support values [20] suboptimal trees up to 15 steps longer were used (per step 10.000 replications and TBR saving 10 trees/rep.; time-out 15 min). Symmetric resampling [21] was performed with 10.000 replications and is displayed as Frequency and GC values.

It was also attempted to do a dating analysis in MrBayes 3.2 [22]. However, unlike the test case for total evidence dating [2], the current data set for Orussidae consists entirely of morphological characters. Even if the fossils included could be placed with reasonable confidence, it was not possible to get reliable age estimates; the variation for some of the deeper nodes was in the range of 300+ Ma in some analyses. There are too few fossils relative to the extant taxa and they are not dispersed evenly across the phylogeny, i.e., three out of four fossils are at or very close to the base of extant orussids, only one is placed more distally in the phylogeny. For these reasons, we do not consider the dating results any further.

## Results

### Redescription of *Baltorussus velteni* Schedl, 2011

Body length 8.9 mm (holotype, Fig. 1), 7.8 mm (non-type, Fig. 2). Holotype covered with film partly obscuring sculpture and setation, making body colour appear whitish green; wing and wing venation appear similarly pale whitish, except for darkened area in fore wing distal to pterostigma; many cracks around specimen, e.g., around frons and along antennae and fore leg obscuring some features. Non-type also with film imparting golden sheen; wing venation dark, fore wing area distal to pterostigma maybe slightly infuscate; distal parts of fore and mid legs cut off through tibia during trimming of amber piece.

#### Head

**Figure 4:**
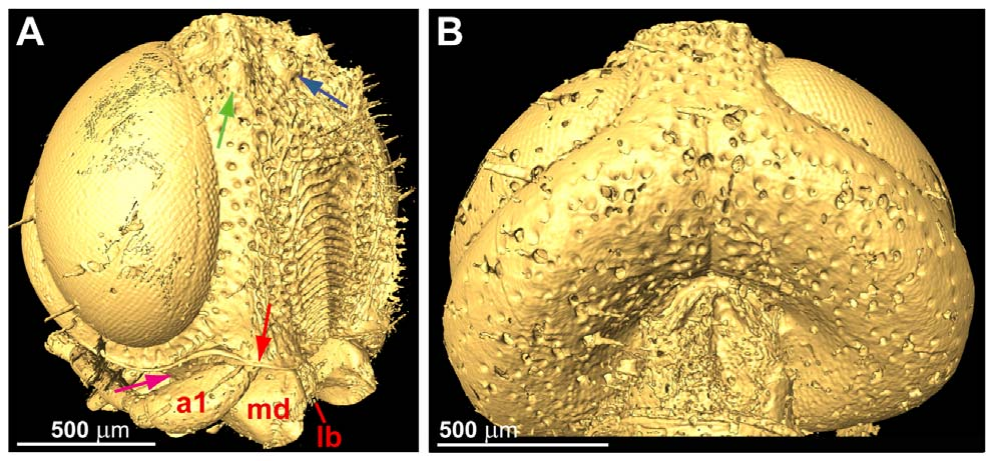
*Baltorussus velteni*, holotype (NHMW 2014/0110/0001), microCT scanning. (A) head laterofrontal view; (B) posterodorsal view. a1  =  first antennomere; lb  =  labrum; md  =  mandible. Green arrow  =  lateral coronal tooth; blue arrow  =  median ocellus; red arrow  =  ventral transverse frontal carina; violet arrow  =  subantennal groove.

**Figure 5:**
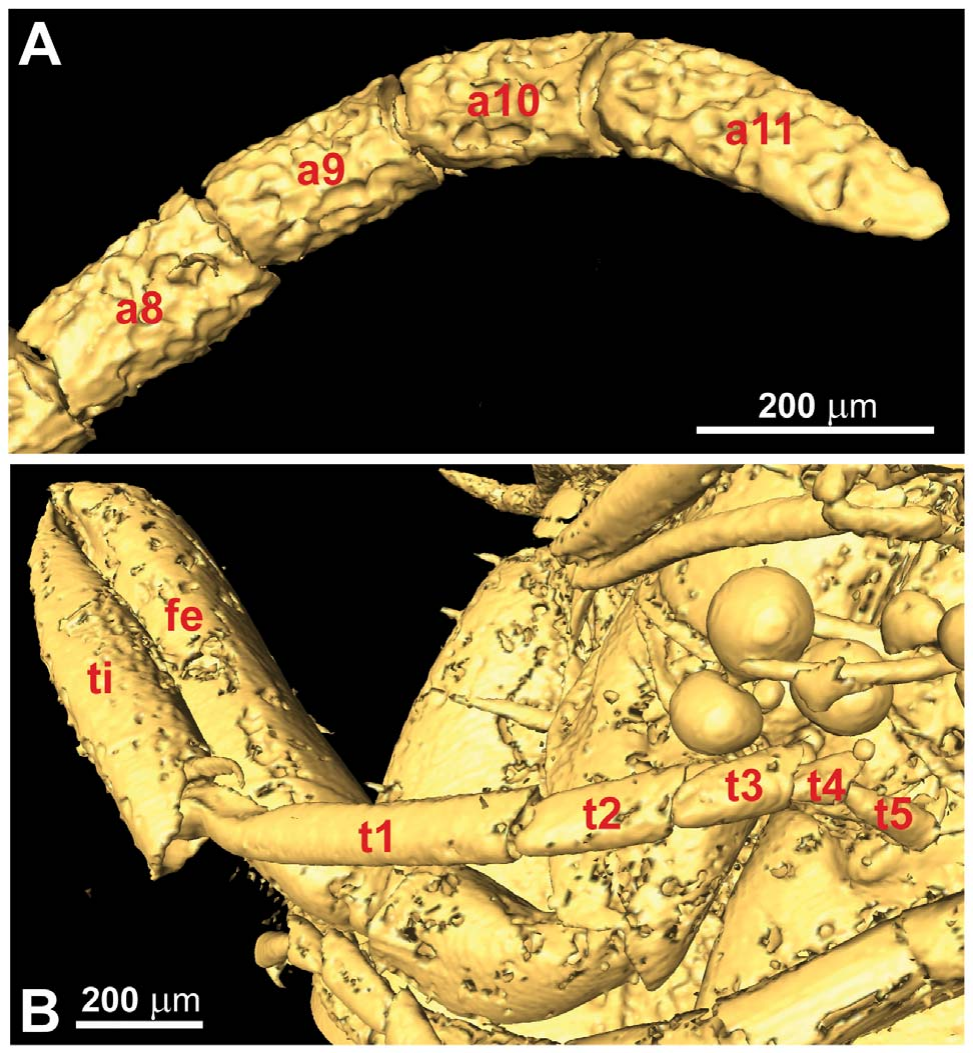
*Baltorussus velteni*, holotype (NHMW 2014/0110/0001). (A) tip of antenna; (B) fore leg. a8-11  =  antennomeres 8-11; fe  =  femur; ti  =  tibia; t1-5  =  tarsomere 1–5.

**Figure 6:**
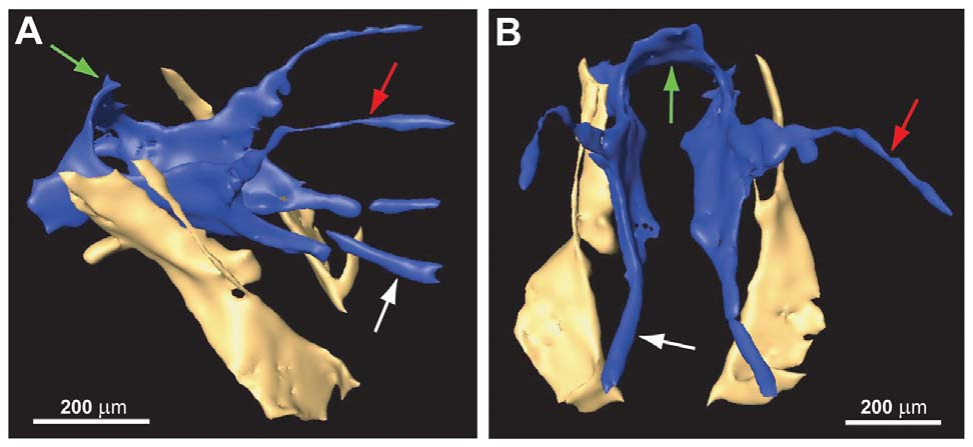
*Baltorussus velteni* holotype (NHMW 2014/0110/0001), tentorium. (A) lateral view (anterior to right) (B) anterior view; tentorium blue, mandibular tendons yellow. Reconstructed from microCT scanning. Green arrow  =  tentorial bridge; red arrow  =  dorsal tentorial arm; white arrow  =  anterior tentorial arm.

Ocellar corona well developed, ventral coronal tooth absent (Figs 3A, B, 4A). Longitudinal frontal carinae absent. Frons with conspicuous sculpture medially, from short dorsal frontal transverse carina ventral to ocellar corona to ventral transverse frontal carina below eyes; sculpture consists of elongate oval area medially with raised, dorsally arching transverse carinae; frontal sculpture otherwise irregularly rugose punctate with irregular short longitudinal carinae laterally; elongate, straight hairs present dorsal and lateral to oval area. Ventral transverse frontal carina not developed medially, not covering antennal bases, laterally continuous with lateral carina of subantennal groove. At most short scattered hairs present posterior to eyes and elsewhere on posterior part of head, sculpture sparsely punctate. Postocular and occipital carina absent (Fig. 4B). Lateral carina of subantennal groove low, terminating level with posterior margin of eye. Antenna with eleven antennomeres, proximal antennomeres slightly expanded distally; scape elongate, cylindrical, slightly curved, pedicellus short; antennomere 10 not expanded, antennomere 11 not modified, 1.5 times longer than antennomere 10 (Fig. 5A). Labrum small, ovoid, with conspicuous hairs ventrally (Figs 3A, B, 4A). Mandibles chisel-like, no teeth developed, internally connected to large flattened tendons extending to level of tentorial bridge (Figs 3C, D, 6). Maxillary palp elongate, five-segmented. Tentorium with deep posterior arms connected posteriorly by arched tentorial bridge, bridge with small apodeme anteriorly (Fig. 6); anterior and dorsal tentorial arms slender, only anterior arms fused with head capsule. Frons internally with unpaired median and paired submedian longitudinal septa along most of its length (Fig. 3C, D).

#### Thorax

**Figure 7:**
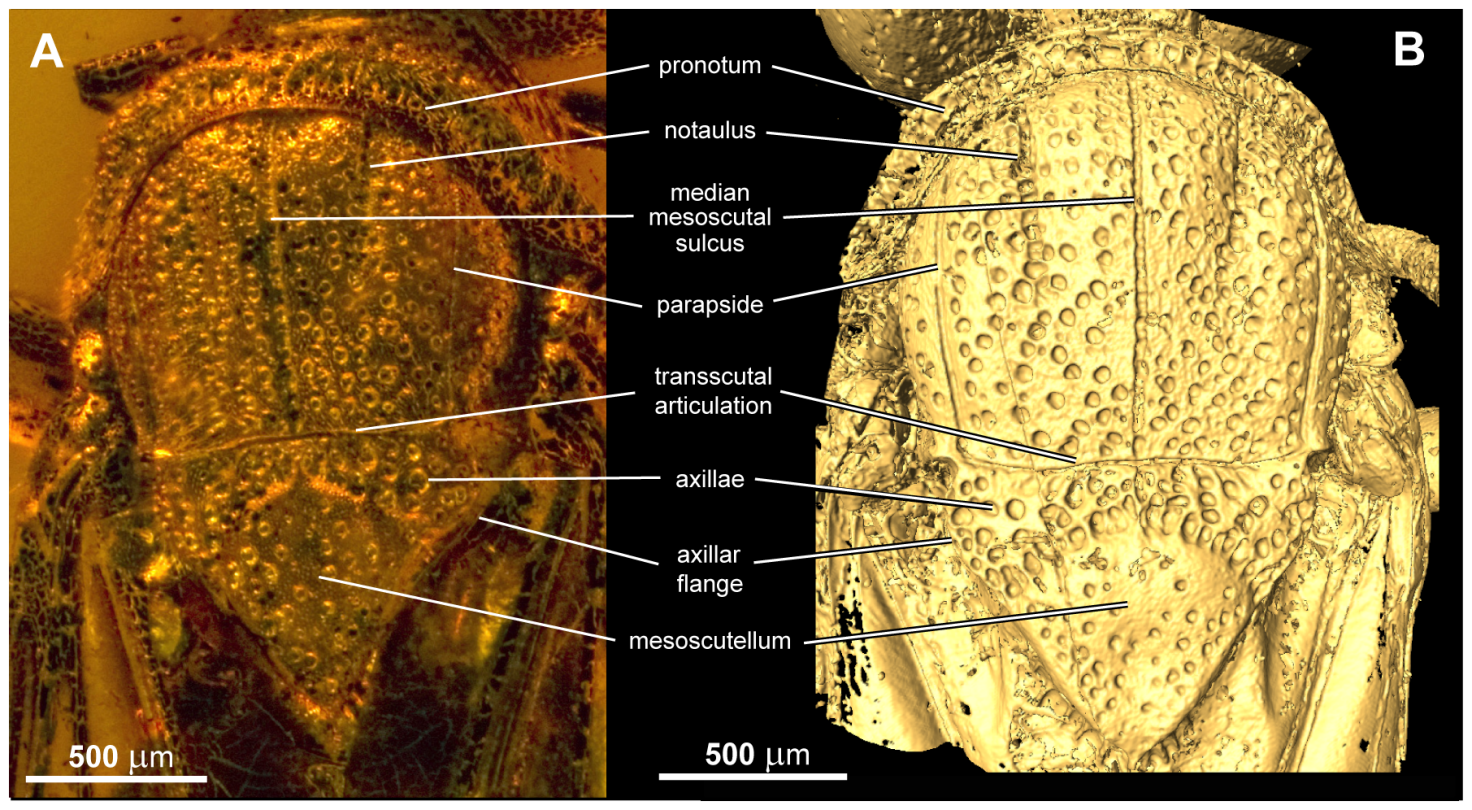
Dorsal view of thorax of *Baltorussus velteni*. (A) non-type (NHMW 2014/0191/0001), brightfield, (B) holotype (NHMW 2014/0110/0001), surface rendering.

Pronotum dorsally of equal length throughout, deeply incurved posteriorly and with glabrous posterior margin, evenly punctate/rugose laterally (Fig. 7). Fore legs slender, tibia not expanded distally or with transverse arching furrow; tarsus five-segmented, basitarsus not modified (Fig. 5B). Mesoscutum sparsely punctate, covered with fine hairs. Mesoscutal sulcus and parapsides conspicuous as narrow furrows, extending to transscutal articulation (Fig. 7); notauli less well defined depressions, extending only halfway to transscutal articulation. Axillae continuous medially, with well developed axillar flanges. Mesoscutellar sulcus well developed, crenulate; mesoscutellum raised, triangular, with well defined lateral margins and acute posterior tip; mesoscutellum with scattered, small punctures (Fig. 7); posterior margin of mesonotum continuous posterior to mesoscutellum. Mesobasalare large, subalar carina well developed, mesepisternal carina absent. Mid coxa subdivided, with distinct lateral carina. Hind coxa with medioventral margins rounded, posterolateral carina well developed. Hind femur without carina and denticles ventrally, posteroventral corner rounded. Hind tibia dorsally with two rows of distinct pegs accommodating hairs; lateral carina absent, ventral carina well developed proximally; apical flange somewhat developed, apical tibial spurs short, unequal in length.

#### Wings

**Figure 8:**
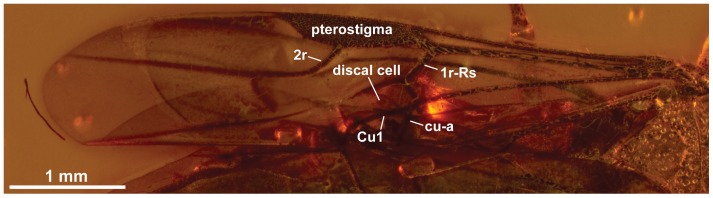
*Baltorussus velteni*, non-type (NHMW 2014/0191/0001), fore wing.

Fore wing vein 1r arises just over halfway from base of pterostigma (Fig. 8); vein 1r-Rs well developed, elongate, discal cell rectangular, proximal part not broader than distal part; vein cu-a inserts on Cu1 slightly distal to M. Hind wing venation cannot be observed.

#### Abdomen

**Figure 9:**
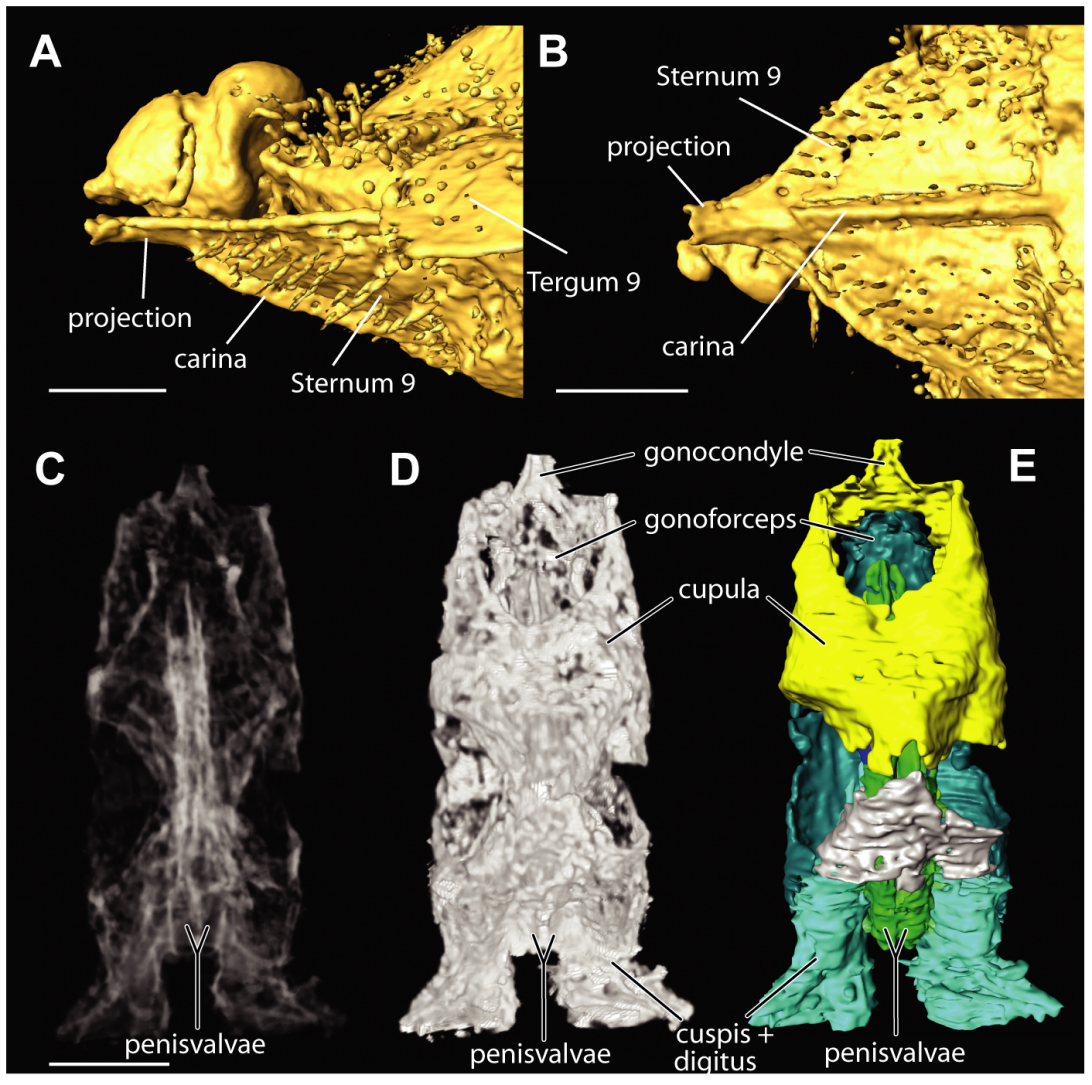
*Baltorussus velteni* holotype (NHMW 2014/0110/0001), abdominal tip, surface rendering. (A) lateral view, (B) ventral view. Male genitalia, dorsal view, anterior/proximal to the top. (C, D) volume renderings; (E) 3D reconstruction. For comparison with extant species of *Orussus*, see [28], fig. 10C-D, and [29], plate 4I-J.

Most of dorsal part of abdomen covered by wings in both specimens. Sculpture of abdomen obscured by artifactual film. No spiracle observed on tergum 8. Sternum 9 triangular in ventral view, with median longitudinal carina bordered by narrow groves extending for ¾ of distance to apex (Fig. 9A, B); apex with prominent truncated projection extending beyond tip of abdomen; sternum 9 otherwise devoid of spines and tubercles. Male genitalia not visible externally. Cupula, gonoforceps and volsella closely integrated (Fig. 9C-E). Cupula strongly emarginate anterodorsally, thin and continuous ventrally, with well developed gonocondyle (apodeme) anteromedially. Gonoforceps grooved and distended anteroventrally, shorter than volsella. Volsella posteriorly differentiated into digitus and cuspis, contiguous to posterior margin of S9; paired penisvalvae approximately as long as gonoforceps.

#### Female

Not known.

### Diagnosis

*Baltorussus velteni* is characterized by the following unique traits that have not been observed in any other living or extinct Orussidae: 1) frons with distinct sculpture in the middle, i.e., a column of transverse dorsally arched carinae forming washboard-like structure (Figs 3A, B, 4A); 2) mesoscutum with well developed mesoscutal sulcus extending from pronotum to transscutal articulation (Fig. 7; some specimens of the extant species *Orussella dentifrons* (Philippi) have a sulcus developed for a short distance anteriorly, but never all the way back to the transscutal articulation); 3) the male sternum 9 with a median longitudinal carina extending for most of its length and distally terminating in a truncated projection (Fig. 9A, B). A similar projection is present in a number of genera within the ‘ophrynopine’ clade, see [7], [23]. However, these taxa never have the median longitudinal carina and in addition to the posterior projection always have anteromedial and lateral spines or tubercles that are absent from *Baltorussus*.

### Results of phylogenetic analyses

**Figure 10:**
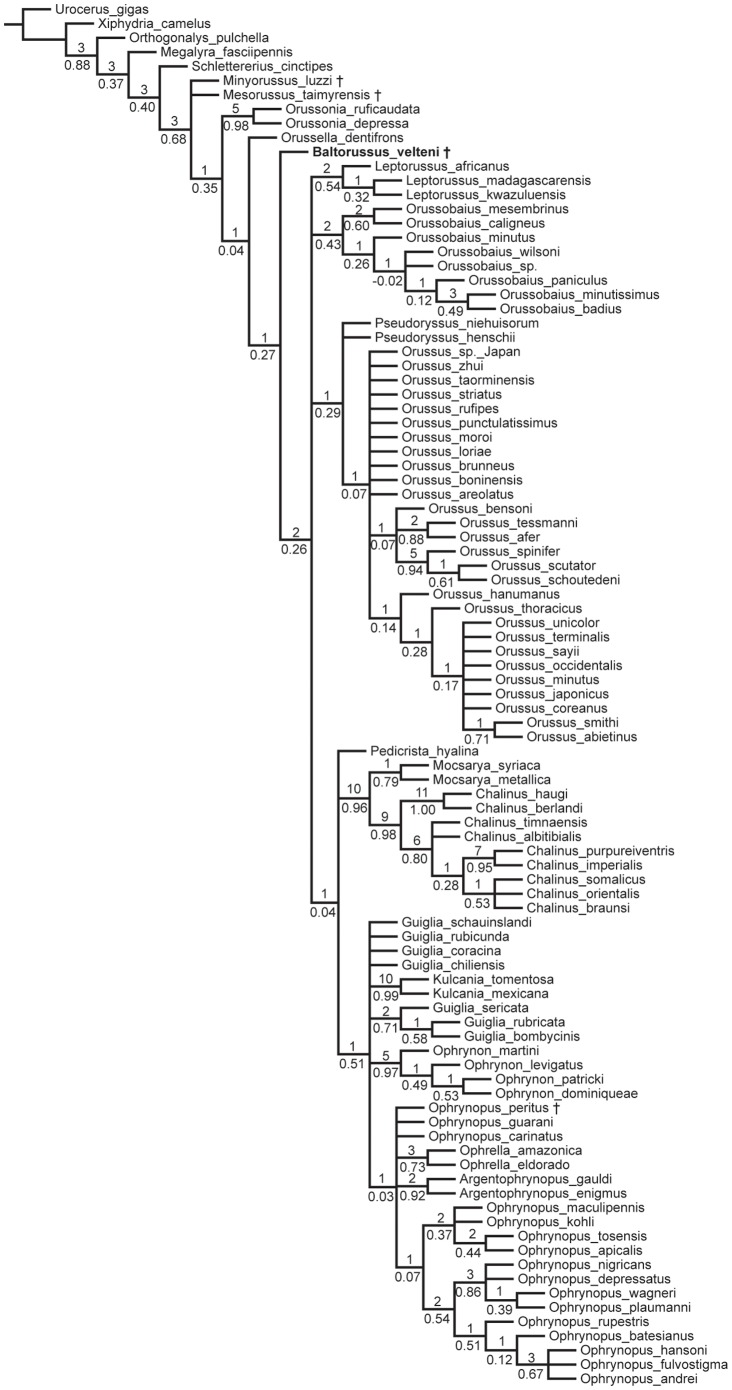
Strict consensus of 20.503 shortest trees (859 steps, CI 0.25, RI 0.766). Bremer support values are displayed above, GC values (group support/contradiction; −1 to 1) of symmetric resampling of 10.000 replicates are displayed below branches. + indicates extinct taxon.

**Figure 11:**
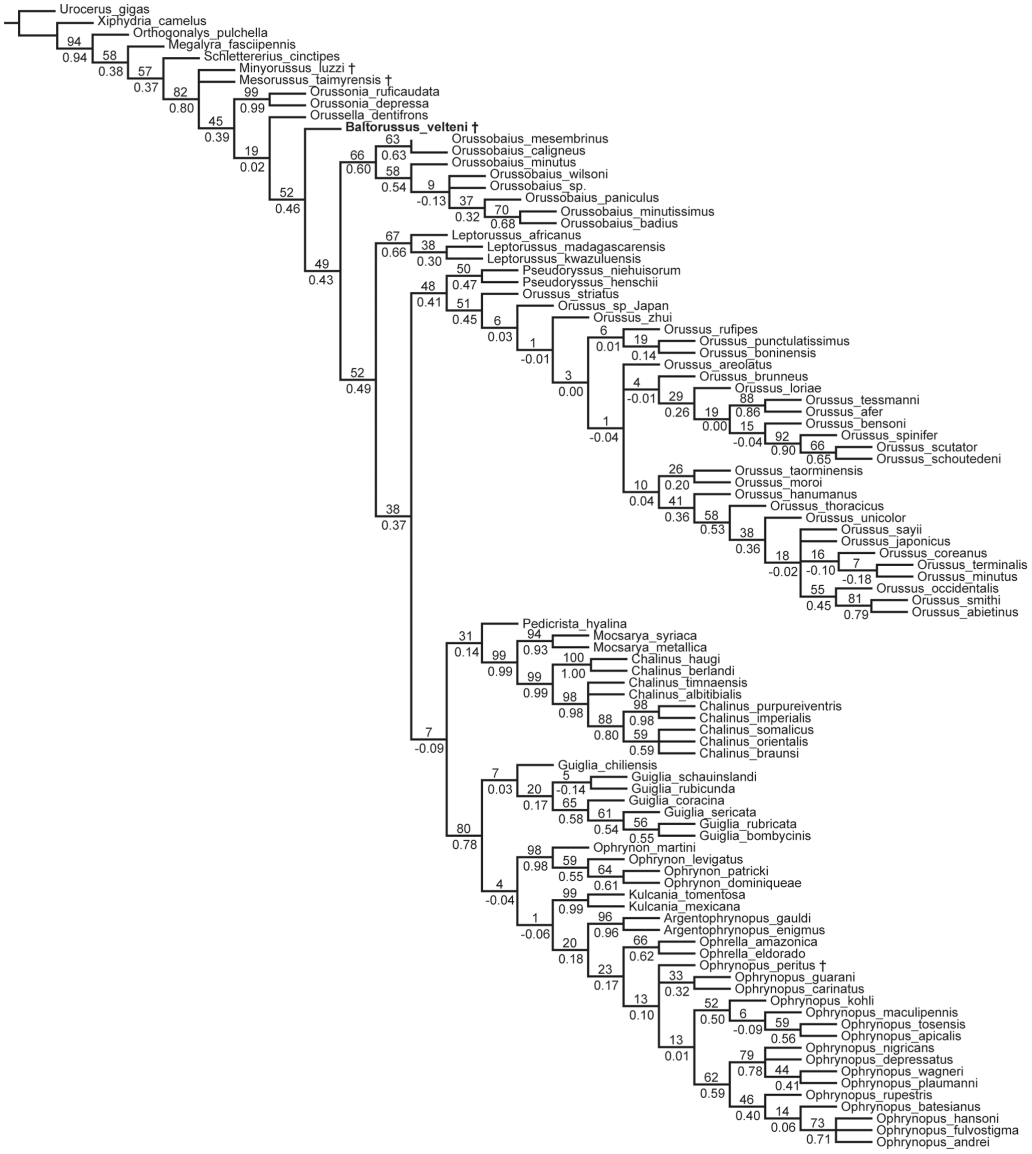
Strict consensus of three equally fit trees retrieved by implied weighting (K = 10–20). Support values were calculated with K = 10. Group frequencies > 50 of symmetric resampling of 10.000 replicates are displayed above, corresponding GC values below branches. + indicates extinct taxon.

The analysis under equal weights yielded 20.503 trees with a length of 859 steps, a consistency index (CI) of 0.25, and a retention index (RI) of 0.766 (Fig. 10). The analyses under implied weighting with K 10–20 consistently produced three trees with a length of 860 and a relative fit ( =  adjusted homoplasy) of 38.89 (Fig. 11). These trees differ only in the relationships within *Orussus*. In all analyses under equal and implied weights *Baltorussus* is placed as a separate lineage being sister to a large clade comprising all extant genera except *Orussonia* and *Orussella*. This position is moderately well supported under equal weights (Fig. 10) and well supported when homoplasious characters are down-weighted through implied weighting (Fig. 11).

## Discussion

### Reinterpretation of the sex and head sculpture

The holotype of *Baltorussus velteni* was originally identified as a female, based on the perceived ovipositor apex protruding from the tip of the abdomen (a common artifact also in pinned extant specimens) and it was stated that the antennae ‘probably’ had ten segments and that the apical one was strongly tapered [10]. These statements fit well with the specimen being a female. However, they differ strongly from our observations in several points:

- The number of antennomeres is in fact 11. The distalmost antennomere is not reduced in size, but of similar width and longer than the penultimate antennomere (compare Fig. 5A with figs 1A–D in [6]), i.e., the antenna is not modified for tapping wood.
- The fore tibia (not observed in [10]) is not swollen and subdivided by a transverse furrow, and the tarsus is five-segmented and does not have an elongate basitarsomere with a projecting spur distally (compare Fig. 5B with figs 2A, C in [6]), i.e., the fore leg is not modified for receiving and processing vibrations reflected from the wood.
- We did not observe a functional spiracle on abdominal tergum 8. Female orussids have functional spiracles on terga 1 and 8, males only on tergum 1.
- We cannot agree that the projection at the apex of the abdomen is the tip of an ovipositor. It is clearly continuous with the posteroventralmost sclerite in the abdomen (Fig. 9A, B), which we interpret as sternum 9 (posteriormost ventral sclerite in the abdomen, absent in female Hymenoptera). The posteroventralmost sclerite in the female abdomen of Orussidae is tergum 9, which is subdivided ventromedially to allow the ovipositor to be exerted. The median longitudinal structure observed on the posteroventralmost sclerite of the abdomen is a carina, not a furrow (Fig. 9A, B).
- The holotype of *Baltorussus* has what appear to be male genitalia inside the posterior end of the abdomen (Fig. 9C–E). All extant Orussidae have the male genitalia concealed when not in use. In the females, the concealed ovipositor apparatus extends all the way into the thorax. No trace of an internalized ovipositor was observed in the microCT scans.

In conclusion there can be no doubt that the holotype of *Baltorussus velteni* is a male. The newly discovered specimen resembles the holotype in all sex specific characters and is also a male.

The sculpture on the anterior part of the head (frons) between the eyes of the holotype of *Baltorussus* was not considered to belong to the specimen in the original description [10]. Instead, it was interpreted as an exuvium, the shed skin of a larve or a pupa of a not further identified insect. Indeed, in the holotype the frons is framed by a rectangle formed of cracks that might give the impression that this area is set apart from the rest of the specimen (Fig. 3A). However, in the microCT scannings performed by us it is evident that the spectacular sculpture is a part of the frons (Figs 3B, 4A). Furthermore, the finding of a second specimen with similar structure (Fig. 2A) confirms that it is indeed an integral part of the head.

The application of microCT scanning to the holotype of *B. velteni* as a supplement to examining the specimen in brightfield microscopy allows us not only to observe internal structures (e.g., the internal head skeleton, the male genitalia) but also to refine the interpretation of the external morphology. Many of the mistakes made in the original description [10] (most notably the misinterpretation of the anterior head sculpture) were apparently due to debris and cracks in the amber matrix and could be corrected by using microCT surface reconstructions. Still, traditional brightfield microscopy remains essential for the investigation of amber fossils as hairs and setae are often insufficiently resolved by microCT data (Fig. 9A, B).

### Phylogenetic position of *Baltorussus*

*Baltorussus* displays a unique combination of plesiomorphic and apomorphic characters that decide its placement within the phylogeny of Orussidae. The plesiomorphic features are (character numbers refer to [7]):

1. The absence of ventral coronal teeth (char. 3:0; Figs 3A, B, 4A). The ventralmost pair of cuticular teeth situated well below the median ocellus is present in most extant Orussidae, where they form the ventral part of the ocellar corona. However, these teeth are absent from the basalmost lineages *Orussonia*, *Orussella*, and *Orussobaius* as well as secondarily in *Mocsarya* and *Pedicrista*.
2. Ventral transverse frontal carina not developed medially (char. 19:1; Figs 3A, B, 4A). Most extant Orussidae have a broad, deep transverse carina extending across the lower part of the head below the eyes and covering the bases of the antennae. This is not the case for *Orussonia*, *Orussella*, and *Orussobaius*, where the carina is less developed and the antennal bases are exposed.
3. Postocular and occipital carinae absent (chars 24:0, 26:0; Fig. 4B). Most orussid genera have these carinae, which are situated just behind the eye or on the back of the head, respectively, partly or fully developed (*Ophrynon* has secondarily lost the occipital carina), but not the genera *Orussonia*, *Orussella*, *Orussobaius* and *Leptorussus*.
4. Dorsal tentorial arms fully developed (Fig. 6). The tentorium forms the internal skeleton of the head and accommodates attachment sites for muscles moving the antennae and mouthparts. Usually, the tentorium has a characteristic Y-shape in lateral view with the posterior tentorial arm forming the ‘stem’ and the diverging anterior and dorsal arms the ‘arms’ of the Y. This is the condition displayed by *Baltorussus* and is certainly a plesiomorphic trait. The presence or absence of the dorsal tentorial arms has not been scored across the Orussidae as it would require destructive dissection or expensive and time consuming scanning procedures. Therefore, the configuration of the tentorium has been documented only for two species of extant Orussidae [24], [25], both within *Orussus*. In this derived genus, the dorsal tentorial arms are much reduced, hardly being developed at all. However, until the internal skeleton of the head of more orussid genera have been examined it cannot be determined when the reduction of the dorsal tentorial arms took place.
5. Median mesoscutal sulcus extending the length of mesoscutum (char. 65:2; Fig. 7). This is the most prominent plesiomorphic feature displayed by *Baltorussus*, as it is not known from any other extant or extinct orussids. This feature is present in most other basal hymenopteran lineages [26], [27] and is probably a retained plesiomorphy that must have been lost independently at least twice among the extant Orussidae. Unfortunately, there is no information on this character from the two Cretaceous fossils, *Mesorussus* and *Minyorussus*.
6. Medioventral margins of hind coxa not angled (char. 96:0). Only *Orussonia* and *Orussella* among extant Orussidae do not have the inner margins of the hind coxa angled.
7. Fore wing venation (chars 115:1; 119:0; 120:1; 123:0; Fig. 8). The fore wing venation of *Baltorussus* conforms completely with what can be inferred to be the ground plan of Orussidae, not displaying any features characteristic for some genera within the family; i.e., vein 2r is not displaced distally on the pterostigma, the discal cell is rectangular and vein 1r-Rs is elongate, and vein cu-a is not displaced significantly distally on Cu1.

On the other hand, there are some traits displayed by *Baltorussus* which are apomorphic relative to the ground plan of the Orussidae:

8. Subantennal groove present, delimited laterally by a short carina (char. 31:1; Fig. 4A). This depression is situated below the eye and accommodates the base of the antenna. It is shallow and not delimited laterally in *Orussonia*, *Orussella*, and *Orussobaius*. *Leptorussus* and *Pedicrista* have a condition similar to *Baltorussus*, whereas all other extant Orussidae have deep grooves delimited laterally by a carina extending well onto the back of the head.
9. Axillar flanges present, distinct (char. 70:1; Fig. 7). These structures are swellings or carinae observed on the posterolateral parts of the mesonotum in all extant Orussidae except *Orussonia*.
10. Lateral carina on mid coxa distinct (char. 87:2). Only *Orussonia* and *Orussella* among extant Orussidae have the mid coxal carina less well developed.
11. Pegs on hind tibia present (char. 103:1). All extant Orussidae except *Orussonia* and *Orussella* have more or less developed pegs dorsally on the hind tibia serving to accommodate prominent hairs.

When analysed with the remainder of the Orussidae, *Baltorussus* is placed inside the extant members of the family as a separate, basal lineage being the sister to a large clade comprising all the extant genera except *Orussonia* and *Orussella* (Figs 10, 11). This is in contrast with the two Cretaceous fossils *Mesorussus* and *Minyorussus*, which are stem group orussids, and *Ophrynopus peritus* from the Miocene, which is placed in an extant genus within the derived ophrynopine clade. There is thus good correlation between the respective ages of the four fossil orussid taxa and their placement in the phylogeny of the family.

## Conclusion: The Timing of the Diversification of the Orussidae

Vilhelmsen [12] explored two sources of evidence when attempting to estimate the age of the radiation of the Orussidae: the direct evidence provided by the fossils and the indirect inferred from correlating splitting events in the family with major tectonic events in Earth history (i.e., biogeography). The age estimates from these two sources differed widely. Since only the two Cretaceous fossils (both from the northern hemisphere) were available for the analyses in Vilhelmsen [12] which placed them both as stem group orussids, they could only provide a minimum age (95 Ma) for the crown group. Consequently, the interpretation of the evolutionary history in Vilhelmsen [12] relied heavily on biogeography.

The four basalmost extant orussid lineages are all from the southern hemisphere in former parts of Gondwana: *Orussonia* (Australia), *Orussella* (Neotropics), *Orussobaius* (Australia), *Leptorussus* (Afrotropical). Based on this distribution, it was concluded that the earliest splitting events within the crown group probably occurred inside Gondwana [12]. Furthermore, the age of some of the splitting events basally in Orussidae between northern and southern hemisphere taxa were interpreted as possible vicariance events correlated with the initial separation between Laurasia and Gondwana (155 Ma ago), later splitting events perhaps corresponding to Gondwanan breakup. This was taken to indicate that the earliest splitting events within Orussidae occurred in the early Mesozoic, i.e., 180 Ma ago. This age estimate infers that the Cretaceous stem group fossils only occurred 80+ Ma after the initial radiation of the Orussidae and thus are poor indicators for the age of the family.

Since the publication of Vilhelmsen [12] the fossil record of Orussidae has been extended into the Paleogene by the discovery of *Baltorussus* and *Ophrynopus peritus*, both of which belong to the crown group. The different ages and phylogenetic placement of these two fossils allow for a better age estimate for the radiation of the family than from the stem group fossils alone. The geographic provenance of *Baltorussus* disproves that the earliest splitting events among extant Orussidae were restricted to southern hemisphere continents. Furthermore, the phylogenetic position and age of *Baltorussus* does not corroborate the biogeographical dating scenario. Having a comparatively young (44 Ma) fossil among the basal extant lineages in combination with the age of the stem group fossils indicates a much later age for the early radiation of Orussidae than indicated by the distributional history.

**Figure 12:**
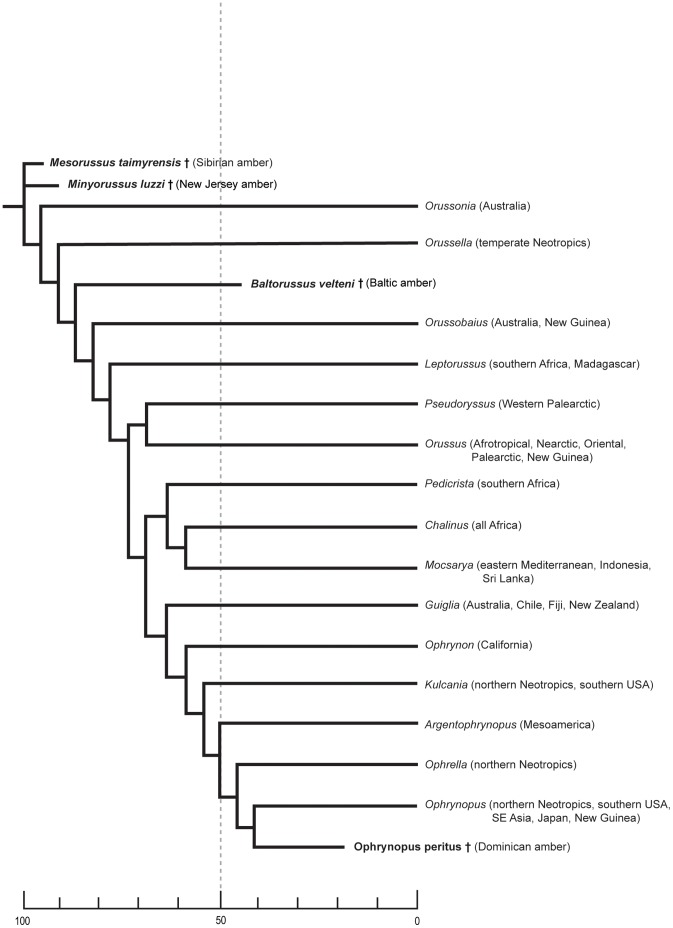
Evolutionary scenario for Orussidae, including fossil taxa. Based on results from implied weights analysis with K = 10–20 (see Fig. 11). Scale indicates age in Ma. Distribution is stated after the terminal. For distribution details of individual species and age and provenance of the Cretaceous fossils, see [12].

If the fossil orussid taxa are assumed to be not much younger than the splitting events that gave rise to them, the crown group Orussidae might not have started to radiate until well into the Cretaceous (Fig. 12). Some of the striking characteristics of the family (e.g., adaptations for vibrational sounding, the concealed ovipositor) may not have accumulated until comparatively shortly before that, i.e., still within the Cretaceous. This is corroborated by the apparent absence of these traits in the Paroryssidae (age up to 158 Ma), which are possibly also related to Orussidae (see Introduction). The sister group to the Orussidae and their closest fossil relatives are the Apocrita which have a fossil record extending back into the Early Jurassic, 180+ Ma ago [1]. Again, the temporal succession of fossil Apocrita, Paroryssidae and Cretaceous orussids is congruent with their phylogenetic placement relative to crown group Orussidae. Inferring that the radiation of extant orussids occurred not until well into the Cretaceous (e.g., after 100 Ma ago) is therefore in better accordance with the fossil record than an Early Jurassic date (180 Ma ago).

The revised sex of the holotype of *Baltorussus velteni* and the adjusted time estimate for the diversification of the Orussidae demonstrates the advances that can be made in understanding insect evolution both by the incorporation of new fossil discoveries in existing data sets, and the application of novel technologies (e.g., microCT-scanning) in the study of insect fossils.
